# Multiscale Simulation of Crack Propagation in Impact-Welded Al_4_Cu_9_ Alloy Based on Cohesive Zone Model

**DOI:** 10.3390/ma18214862

**Published:** 2025-10-23

**Authors:** Rongqing Luo, Dingjun Xiao, Guangzhao Pei, Haixia Yan, Sen Han, Jiajie Jiang, Miaomiao Zhang

**Affiliations:** School of Environment and Resource, Southwest University of Science and Technology, Mianyang 621010, China; luorq@mails.swust.edu.cn (R.L.);

**Keywords:** Al_4_Cu_9_ alloy, cohesive zone model, multiscale, molecular dynamics, finite element

## Abstract

The fracture behavior of the Al_4_Cu_9_ intermetallic compound at the interface of impact-welded Cu/Al joints remains insufficiently explored through integrated multiscale modeling and experimental validation. In this study, molecular dynamic (MD) simulations, finite element (FE) analysis implemented in ABAQUS (version 2020) and a cohesive zone model (CZM) were combined with optical microscopy (OM) and scanning electron microscopy (SEM) observations of the interface and crack initiation zones in impact-welded Cu/Al specimens to investigate crack propagation mechanisms under different defect configurations. The experimental specimens consisted of 1060 aluminum (Al) and oxygen-free high-conductivity (OFHC) copper, fabricated via impact welding and subsequently annealed at 250 °C for 100 h. The interfacial morphology and crack initiation features obtained from OM and SEM provided direct validation for the traction–separation (T-S) parameters extracted from MD and mapped into the FE model. The results indicate that composite defects (blunt crack + void) cause a significantly greater reduction in fracture energy and stress intensity factor than single defects and that defect effects outweigh temperature effects within the range of 200–500 K. The experimentally observed crack initiation locations were in strong agreement with simulation predictions. This integrated simulation–experiment approach not only elucidates the multiscale fracture mechanisms of the Al_4_Cu_9_ interface but also provides a physically validated basis for the reliability assessment and optimization of aerospace Cu/Al welded structures.

## 1. Introduction

In the aerospace sector, copper-aluminum alloys have emerged as pivotal materials for conductive cable systems due to their lightweight nature, high conductivity, and robust corrosion resistance [1,2,3]. As aircraft evolve towards lighter and higher-performance designs, the demand for copper-aluminum alloys extends beyond traditional conductive roles, necessitating new alloy systems that offer both high strength and excellent ductility [4,5,6]. However, the disparity in lattice structures between copper and aluminum can lead to the formation of intermetallic compounds during impact welding, which, under external stress, may result in crack formation. The propagation of these cracks not only compromises the mechanical properties and structural integrity of the materials but also poses risks to the safety and reliability of aerospace vehicles. During the annealing process post-high-velocity impact bonding, thermal treatment can induce interface element diffusion and phase transformation, potentially leading to the formation of a stable Al_4_Cu_9_ intermetallic phase [7,8,9,10,11,12,13]. This compound exhibits superior toughness and corrosion resistance, making it more suitable as an interface bonding phase compared to other copper-aluminum compounds [10,14,15,16]. Therefore, understanding and predicting crack propagation in Al_4_Cu_9_ alloys are crucial for ensuring the safe operation of aerospace electrical systems. Traditional experimental methods are limited by spatial and temporal resolution constraints, hindering the capture of atomic-scale crack evolution mechanisms; single-scale numerical simulations fail to effectively correlate micro-defect evolution with macro-fracture behavior [17].

Recently, multi-scale modeling approaches based on atomic cohesion models [18,19] have provided new avenues for elucidating material damage mechanisms. Research indicates that the cohesive zone model effectively characterizes crack propagation across mesoscopic and macroscopic scales [20]. Tvergaard and Hutchinson [21] introduced a cohesive zone model with traction-separation laws using the continuous interface separation constitutive relationship of internal state variable theory. This model is capable of predicting transgranular and intergranular fractures [22]. However, quantifying the T-S law through experiments remains challenging, as related parameters are primarily determined through unreliable assumptions and nanocrystal experiments.

Scholars have discovered that constructing atomic-scale microstructural details and mechanical models [23,24,25] can yield effective T-S curves, making atomic simulations a valuable tool for quantifying the T-S law. In this context, molecular dynamic simulations have emerged as a promising method for obtaining T-S parameters. Recently, the use of the Cohesive Zone Model (CZM) to bridge molecular dynamics and finite element methods for studying material mechanical damage behavior has gained attention [19,26,27,28,29]. Yu et al. [30] developed a cross-scale method using the CZM as a bridge, obtaining CZM parameters for ferritic pearlite steel damage regions through molecular dynamic (MD) simulations and applying them within a finite element (FE) framework to simulate the mechanical damage behavior of ferritic-pearlite steel. Dandekar et al. [31] conducted molecular dynamic simulations to derive the traction-separation law for aluminum-silicon carbide composite systems and incorporated the parameterized traction-separation law into finite element simulations to predict the stress–strain response of metal matrix composites under high strain rate loading.

Against this background, the present study develops a multiscale crack propagation analysis framework for the Al_4_Cu_9_ phase at the interface of impact-welded Cu/Al joints, based on the integration of molecular dynamics (MD), finite element (FE) analysis, and a cohesive zone model (CZM). At the microscale, MD models containing various defect configurations were constructed using the embedded atom method (EAM) to simulate crack propagation processes and to extract traction–separation (T-S) parameters. At the macroscale, these parameters were mapped onto zero-thickness cohesive elements within an FE model implemented in ABAQUS, thereby transferring fracture characteristics from the atomic scale to the structural scale. In addition, temperature-dependent MD simulations in the range of 200–500 K were conducted to elucidate the influence of temperature on dislocation motion and crack path evolution. For experimental support, 1060 aluminum (Al) and oxygen-free high-conductivity (OFHC) copper specimens were fabricated via impact welding and subsequently annealed at 250 °C for 100 h. Optical microscopy (OM) and scanning electron microscopy (SEM) were employed to characterize the interfacial morphology and crack initiation zones after tensile testing, providing essential input for defining defect types and geometries in the microscale models. This combined approach enables the coherent integration of experimental observations, atomistic simulations, and structural-scale analyses, offering mechanistic insights into the multiscale fracture behavior of Al_4_Cu_9_ under various defect and temperature conditions.

Compared with existing multiscale studies (e.g., Dandekar and Shin, 2011 [31] on Al–SiC composites; Yu et al., 2024 [30] on ferritic–cementite steels), the present work offers two key innovations. First, it systematically extends the MD–CZM–FE coupling methodology to the Al_4_Cu_9_ intermetallic compound formed during impact welding, a critical phase that has rarely been investigated from a multiscale perspective. Second, it explicitly quantifies the relative influence of micro-defects and temperature on fracture toughness, thereby revealing the competing damage mechanisms that govern the reliability of Cu/Al welded joints. These contributions highlight the distinctiveness and broader applicability of the proposed framework beyond existing approaches. Importantly, this study integrates experimental investigations to reinforce the physical basis of the modeling. Impact-welded Cu/Al specimens were fabricated, and the interfacial morphology was characterized by OM and SEM to define representative defect types and geometries for the MD models. In addition, tensile tests were performed on the welded joints, and the measured fracture behavior was compared with simulation predictions, ensuring that the multiscale framework is supported by both computational and experimental evidence.

## 2. Materials and Methods

### 2.1. Welding Materials and Technology

**Figure 1:**
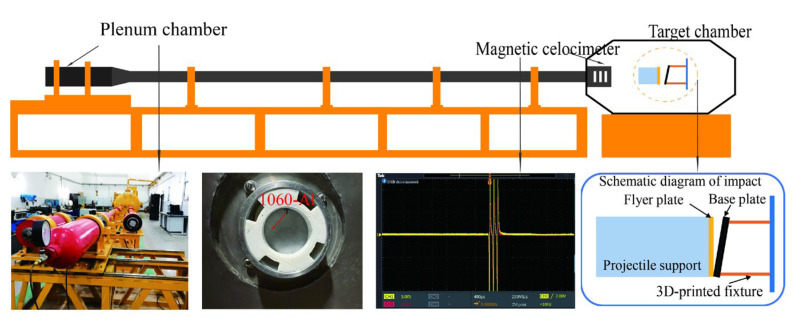
Schematic diagram of the single-stage light gas gun system used for high-velocity impact bonding experiments.

All bonding experiments were performed by high-velocity impact bonding using a single-stage light-gas gun. The apparatus comprises a high-pressure driving chamber, a 37 mm caliber launch tube, and a Ø2 m × 5 m target chamber, as schematically illustrated in Figure 1.

**Figure 2:**
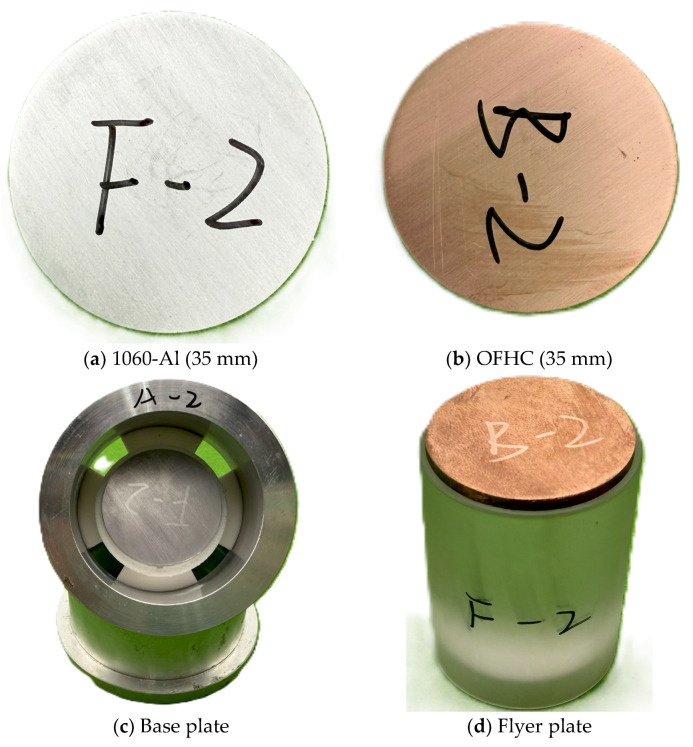
Experimental materials.

To characterize the interfacial structure of Al_4_Cu_9_, commercially supplied 1060 pure aluminum and oxygen-free high-conductivity (OFHC) copper were used as the substrate and flyer, respectively, to fabricate impact-bonded specimens. The 1060 aluminum disks (grade 1060, purity ≥ 99.6%, GB/T 3190-2020 standard [32], Ø35 mm × 2 mm) were supplied by Shenzhen Ruihe Metal Co., Ltd. (Shenzhen, China). The OFHC copper disks (grade TU1, purity ≥ 99.97%, GB/T 5231-2012 standard [33], Ø35 mm × 3 mm) were purchased from Dongguan Guangfeng Metal Materials Co., Ltd. (Dongguan, China) (see specimen dimensions in Figure 2).

The flyer was accelerated to a velocity of 459 m/s with an impact angle of 12°. The surfaces of both 1060-Al and OFHC Cu specimens were ground using 800–2000 grit sandpaper to ensure adequate surface quality. The initial standoff distance between the flyer and the target was not measured or controlled during the experiment.

### 2.2. Experimental Characterization

The bonded Cu/Al specimens were examined to characterize the interfacial morphology. Optical microscopy (OM, Olympus DSX510, Olympus Corporation, Tokyo, Japan) and scanning electron microscopy (SEM, Hitachi SU8600, Hitachi High-Technologies Corporation, Tokyo, Japan) were used to observe the Cu/Al interface. Tensile tests were conducted on a microcomputer-controlled electronic universal testing machine (model CMT5504, Shenzhen SANS Testing Machine Co., Ltd., Shenzhen, China).

**Figure 3:**
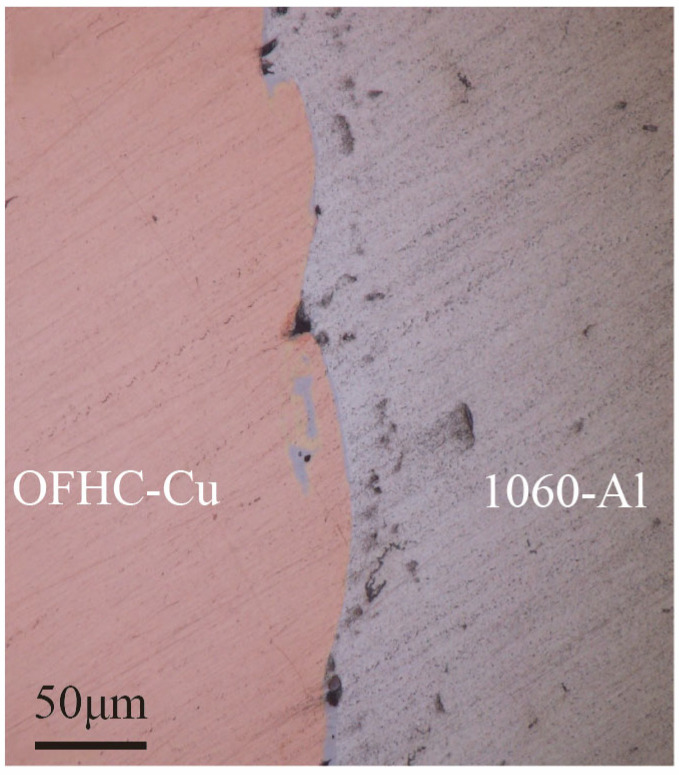
Interfacial morphologies of the 1060-Al/OFHC high-velocity impact bonded composite observed by optical microscopy.

Previous studies [7] have shown that the intermetallic compound (IMC) layer at the Cu/Al interface evolves during annealing: after treatment at 250 °C for 100 h, Al is completely consumed, CuAl_2_ transforms into Al_4_Cu_9_, and Al_4_Cu_9_ becomes the terminal phase. Following the same annealing treatment, the welded samples were examined by optical microscopy, and the Cu/Al interfacial morphology is presented in Figure 3. The results reveal a wavy interface accompanied by localized melted regions, with tight overall bonding and no apparent cracks or voids. Two characteristic features were observed in the vicinity of the interface: localized melted zones and vortex-like structures. The melted zones exhibited two distributions: some connected to the Al matrix, while others were fully enclosed by vortices. These regions are presumed to correspond to brittle intermetallic phases.

**Figure 4:**
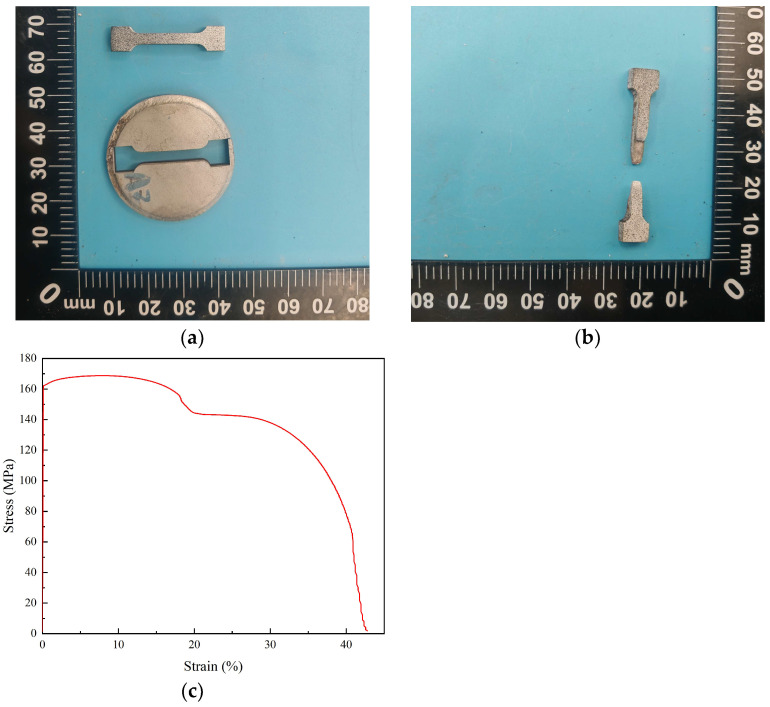
Specimens for uniaxial tensile test and the representative stress–strain curves along the weld interface. (**a**) before test; (**b**) after testing; (**c**) representative stress–strain curve along the weld interface.

The specimens before and after uniaxial tensile testing are shown in Figure 4a,b, respectively. Representative stress–strain curves obtained along the weld interface (parallel to the Cu/Al interface) are presented in Figure 4c. Uniaxial tensile tests were conducted along the plane of the weld interface using a microcomputer-controlled electronic universal testing machine (model CMT5504, Shenzhen SANS Testing Machine Co., Ltd., Shenzhen, China) under quasi-static, displacement-controlled conditions at room temperature (25 °C). These experimental results provide quantitative information on the mechanical behavior of the weld interface and serve as reference data for subsequent molecular dynamic simulations and multiscale modeling.

**Figure 5:**
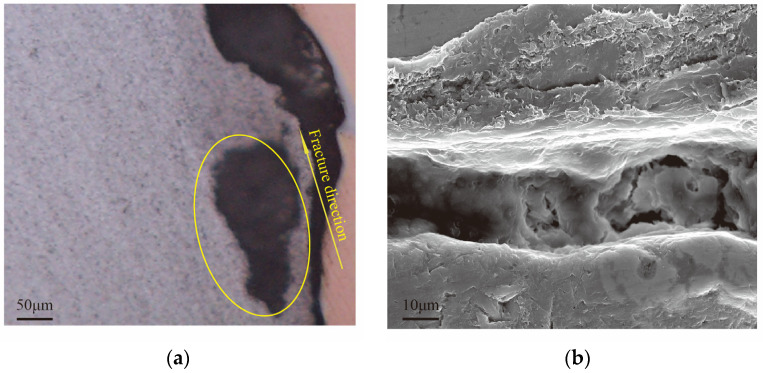
Microstructure of the crack initiation region on the Al alloy side of the tensile specimen: (**a**) OM cross-section showing crack propagation along brittle phase/Al interfaces; (**b**) SEM image with typical brittle fracture features.

**Figure 6:**
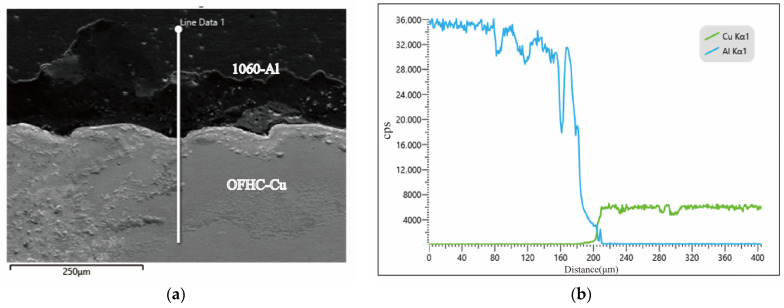
SEM images of the Cu/Al interface in the 1060-Al/OFHC Cu high-velocity impact bonded composite: (**a**) local macrostructure of the interface; (**b**) EDS line scan along the white line indicated in (**a**).

The microstructure of the crack initiation region is presented in Figure 5. Cracks preferentially propagated along the interfaces between brittle phases and the Al matrix, following the overall fracture path. SEM observations further revealed a high density of second-phase particles and typical features of brittle fracture, indicating that intergranular fracture was the dominant failure mode. Elemental distributions across the Cu/Al interface were further examined using EDS line scanning, as shown in Figure 6. The results reveal the variation in Al and Cu concentrations along the interface, providing quantitative insight into the interfacial composition. These data support the construction of realistic atomic models in subsequent molecular dynamic simulations. These findings confirm that brittle intermetallic compounds severely degrade the mechanical integrity of the joints.

To gain atomic-scale insights into the intrinsic fracture mechanisms of Al_4_Cu_9_, a multiscale modeling framework combining molecular dynamics (MD) and finite element (FE) methods was adopted. Three representative atomic configurations were constructed: (1) a defect-free crystal, (2) a crystal containing a pre-existing large crack, and (3) a crack-containing model with an additional void at its center. The tensile fracture behaviors of Al_4_Cu_9_ under these microstructural conditions were systematically simulated, thereby elucidating the role of brittle phases in the fracture process of high-velocity impact bonded Cu/Al joints.

### 2.3. Atomic-Scale Model Construction

#### 2.3.1. Selection and Validation of Potential Functions

The interatomic interactions within materials are described by potential functions, with the selection of an appropriate potential depending on the material type and simulation requirements. Potential functions, which describe the interatomic interactions, significantly influence not only the mechanical behavior of materials but also the simulation accuracy and computational efficiency. Many-body potentials can effectively account for the cooperative effects of surrounding atoms on a target atom. Based on this characteristic, this study employs the embedded atom method (EAM) potential [34], which describes the interatomic interactions in metals and alloys by introducing the energy required to embed an atom’s nucleus in the electron cloud background.

To verify the reliability of the employed EAM/alloy potential function and its associated parameters, a molecular dynamics (MD) model of Al_4_Cu_9_ was constructed. A unit cell was established with crystallographic orientations along X: [1 0 0], Y: [0 1 0], and Z: [0 0 1], under periodic boundary conditions (P P P). Based on this model, the fundamental mechanical properties of Al_4_Cu_9_, including the lattice constant, elastic constants, and cohesive energy, were systematically investigated.

**Figure 7:**
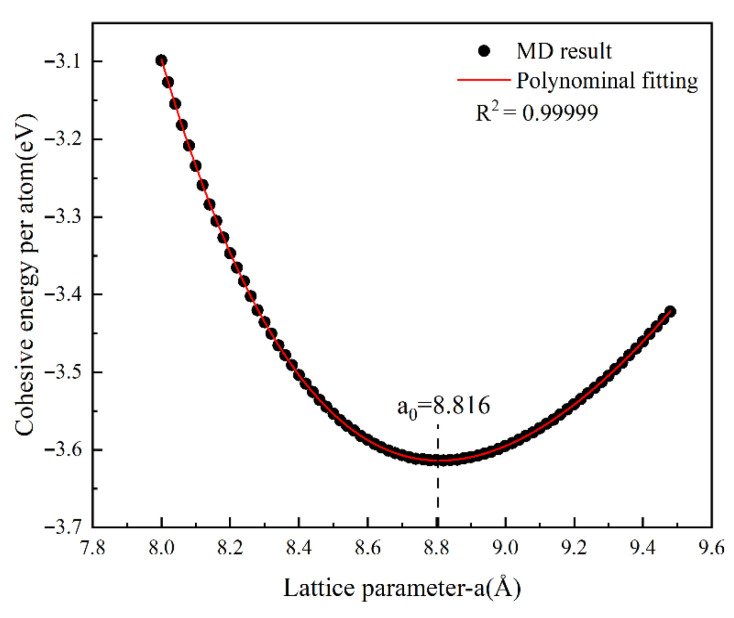
Cohesive energy–lattice constant relationship for Al_4_Cu_9_ and the corresponding polynomial fitting curve.

The cohesive energy was calculated over a range of lattice constants, and the resulting cohesive energy–lattice constant curve is presented in Figure 7. The data were fitted with a fourth-order polynomial, expressed as:

(1) $$E=0.075216{a_{0}}^{4}-2.87157{a_{0}}^{3}+41.60061{a_{0}}^{2}-270.10536a_{0}+655.8799$$

Differentiating Equation (1) and setting its derivative to zero yields the equilibrium lattice constant $a_{0}$. Substituting $a_{0}$ into Equation (1) gives the corresponding equilibrium cohesive energy E.

The bulk modulus was further derived from the following relation:

(2) $$B_{0}=\frac{M}{9V_{0}}{\left.\frac{d^{2}E}{da_{0}^{2}}\right|}_{a_{0}}$$

where M=52 is the number of atoms in the unit cell, and $V_{0}$ is the equilibrium volume of the unit cell.

In addition, based on the generalized Hooke’s law, the elastic constants $C_{11}$, $C_{12}$, and $C_{44}$ were calculated and subsequently compared with experimental values reported in the literature. The results are summarized in Table 1, demonstrating the validity of the employed potential function in reproducing the key mechanical properties of Al_4_Cu_9_.

#### 2.3.2. Construction of the Molecular Dynamics Model

**Figure 8:**
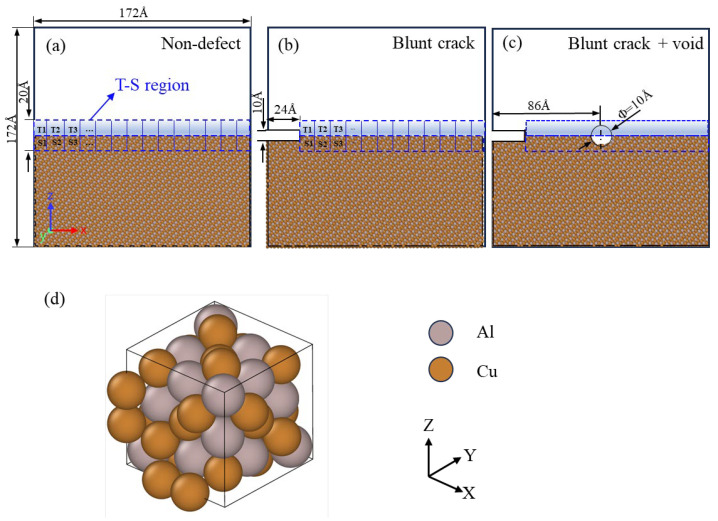
Molecular dynamics (MD) model construction of Al_4_Cu_9_ alloy under different defect configurations: (**a**) Non defect; (**b**) Blunt crack; (**c**) Blunt crack with central void; (**d**) Crystal Structure and Atomic Arrangement of Al_4_Cu_9_ Alloy [39].

The initial model adopts a rectangular structure to eliminate size effects, with a thickness in the Z-direction exceeding four times the lattice constant [36]. Key MD simulation parameters—including the embedded atom method (EAM) potential type, periodic boundary conditions (PBCs) for thermal relaxation, model dimensions (172 Å × 86 Å × 172 Å, 208,000 atoms), and prefabricated crack geometry—are fully specified here to ensure reproducibility. The model is constructed along the [100], [010], and [001] crystallographic directions, with dimensions of 172 Å × 86 Å × 172 Å, comprising 208,000 atoms. As illustrated in Figure 8, an initial crack is prefabricated on the left side of the model, with relevant dimensions marked.

The crack propagation simulation proceeds in two stages: the first stage involves thermal relaxation using periodic boundary conditions (PBCs) and an isothermal-isobaric ensemble (NPT) to achieve thermodynamic equilibrium; the second stage involves tensile loading using a microcanonical ensemble (NVE) to simulate adiabatic conditions. Previous research [37] suggests that a strain rate of 10^−9^/s is appropriate for quasi-static loading. Therefore, the loading conditions involve stretching the upper boundary along the Z-axis at a rate of 0.005 Å/ps, with a linear gradient distribution in the middle region. The total tensile steps number 50,000, with a time step of 0.001 ps, and results are output every 200 steps. These loading parameters, combined with the previously defined model and boundary conditions, provide a complete description of the simulation setup. Atomistic simulations of the above MD model were performed using the LAMMPS (64-bit 17Apr2024-MSMPI) from Sandia National Laboratories [38].

#### 2.3.3. Comparison Between MD Simulation and Experimental Results

To evaluate the consistency between molecular dynamic (MD) simulations and experimental data, the bulk modulus of Al_4_Cu_9_ obtained from MD calculations (*K* = 153.1 GPa) was compared with the experimentally measured Young’s modulus (*E* = 142.5 GPa, converted from 142,485 MPa). The results indicate that the MD-predicted elastic modulus is significantly higher than the experimental value. This discrepancy mainly arises from microcracks, voids, and grain boundaries in the experimental samples, whereas the MD simulations are based on an ideal crystal structure without such defects. Moreover, in the Cu–Al impact-welded material, Al_4_Cu_9_ is only formed in the interfacial region, leading to a lower overall stiffness compared with an ideal single-phase crystal.

Despite these differences, the high-precision elastic parameters obtained from MD simulations can still serve as valuable input for finite element modeling of welded joints, facilitating the analysis of crack propagation paths and critical stress variations, and providing theoretical insights into interfacial mechanical behavior.

### 2.4. Development of the Macroscopic-Scale Model

To explore the impact of atomic-scale evolution on macroscopic failure behavior, this section constructs a compact tension (CT) specimen model with cohesive elements to simulate crack propagation under displacement loading. In this study, cohesive elements are pre-placed along the anticipated crack path, and a predefined cohesive failure approach is adopted. That is, the crack path is fixed, and crack propagation occurs along the preset route. This method ensures consistency between the finite element simulation results and the T-S curves derived from molecular dynamics (MD) while facilitating validation of the computational model. The consistency of the molecular dynamics-derived cohesive zone law (T-S curve) with linear elastic fracture mechanics theory is verified.

**Figure 9:**
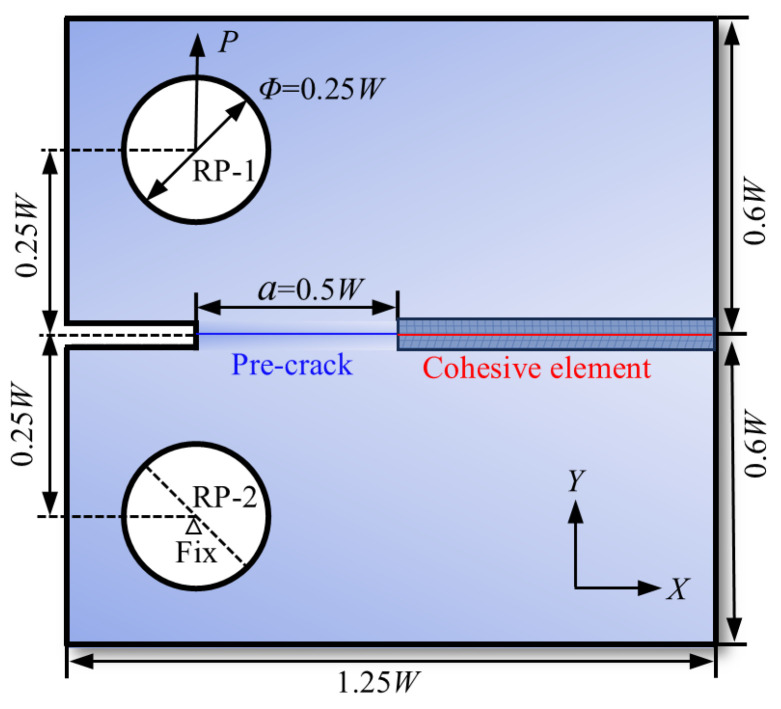
Schematic diagram of CT specimen for FEM modeling.

In this section, the CT specimen geometry with W = 400 μm is designed following the ASTM E399 [40] standard. The specimen width and height are 1.25 W and 1.2 W, respectively, and the hole diameter is 0.25 W (100 μm). A crack with an initial length of *a* = 0.5 W is pre-placed on the left side of the specimen, with cohesive elements arranged along the anticipated crack propagation path. Figure 9 illustrates the FE model of the alloy. The overall FE model employs CPE4R elements. Subsequently, four-node two-dimensional cohesive elements (COH2D4) are embedded in the potential damage zone.

The mesh is refined near the crack tip to accurately capture stress gradients, while coarser elements are used in regions away from the crack. For the CT specimen, the average CPE4R element size is approximately 15 μm, and the minimum cohesive element size along the crack path is 2.61 μm. Attempts to further refine the cohesive elements caused convergence issues, so quantitative mesh comparison could not be performed. Based on finite element simulation experience, this mesh discretization is sufficient to capture crack propagation paths and reaction force variations, indicating that the mesh has a minor impact on the main results and ensures numerical stability and reliability.

The reference point RP-2 is fully constrained, and displacement is applied to RP-1 to drive crack propagation; reaction forces are extracted at RP-1 for load–displacement analysis. Similar mesh density control strategies have been employed in other numerical analyses of linear elastic fracture mechanics [41].

### 2.5. Development of the Macro-Micro-Scale Cohesive Zone Model

The cohesive zone finite element method is employed to simulate crack propagation, assuming that the crack surface is subjected to normal separation and tangential slip traction forces, which are closely related to displacement. Crack evolution is characterized by three elements: initial stiffness, damage initiation criterion, and damage evolution law. Investigating the relationship between traction (T) and displacement (δ) (T-S law) is central to revealing the damage evolution mechanism. Abaqus provides exponential and bilinear constitutive models, with the bilinear model being more suitable for brittle fracture analysis. Key parameters include initial displacement ($\delta_{\mathrm{ini}}$), maximum interface traction ($N_{\max}$), failure displacement ($\delta_{fai}$), and interface fracture energy ($G_{c}$):

(3) $$G_{c}=\frac{1}{2}N_{\max}\times \delta_{fai}$$

The initial stiffness K can be calculated from the damage initiation point on the bilinear T-S curve, as follows [26]:

(4) $$K=\frac{N_{\max}}{\delta_{\mathrm{ini}}}$$

In Mode I fracture, the material is isotropic with equal normal and tangential stiffness. The extended formula is:

(5) $$K=K_{n}=K_{s}=K_{t}=\frac{N_{\max}}{\delta_{\mathrm{ini}}}$$

Based on previous studies, a reasonable T-S statistical region width is 20 Å [42], and the T-S region length should be six times the initial crack length [43]. This study selects a 148 Å × 20 Å region for measurement.

In the molecular dynamic model, the T-S region is defined as specific atomic layers above and below the crack surface (see the blue dashed region in Figure 8). The crack opening displacement (δ) is obtained by calculating the average displacement difference in atoms in this region, while the average atomic stress characterizes the interface traction (T). Only data from the T-S region is used to plot the curve, ensuring result consistency.

To clarify how the energy and traction parameters obtained from molecular dynamics are implemented in ABAQUS and their unit conversions, the corresponding relationships are summarized as shown in Table 2.

### 2.6. MD–FE Multiscale Coupling Strategy and Implementation

A multiscale coupling framework integrating molecular dynamics (MD), finite element (FE) analysis, and a cohesive zone model (CZM) was established to enable the effective transfer of atomistic-scale fracture characteristics to structural-scale mechanical responses. In the MD simulations, Al_4_Cu_9_ interfacial models containing various defect configurations were constructed using the embedded atom method (EAM) potential. Under uniaxial tensile loading, the traction–separation (T-S) response was obtained by averaging the relative displacement and virial stress of atoms within a predefined T-S region. The resulting data were smoothed and fitted to a bilinear cohesive law to extract key parameters, including the maximum interface traction ($N_{\max}$), fracture energy ($G_{c}$), initial displacement ($\delta_{\mathrm{ini}}$), and failure displacement ($\delta_{fai}$). These parameters were converted to engineering units and used to calculate the initial stiffness (*K*) from the elastic slope of the T-S curve. The processed cohesive parameters were then assigned to zero-thickness two-dimensional cohesive elements (COH2D4) embedded along the predefined crack path in the ABAQUS FE model, with the bilinear CZM formulation selected to ensure consistency with the MD-derived T-S law. The MD-derived cohesive properties were assumed to be intrinsic to the interface and thus scale-independent; geometric scaling was applied to match the compact tension (CT) specimen dimensions while preserving the energy release rate and peak traction. Experimentally, 1060 aluminum (Al) and oxygen-free high-conductivity (OFHC) copper specimens were fabricated via impact welding and annealed at 250 °C for 100 h. Optical microscopy (OM) and scanning electron microscopy (SEM) were employed to characterize the interfacial morphology and crack initiation zones after tensile testing, providing the defect types and geometries used in the MD models. The crack initiation locations predicted by the FE simulations were compared with experimental observations to validate the proposed coupling approach.

## 3. Results

### 3.1. Analysis of Crack Propagation Behavior Under Different Defect Conditions

#### 3.1.1. Microscale Fracture Simulation Under Different Defect Conditions

**Figure 10:**
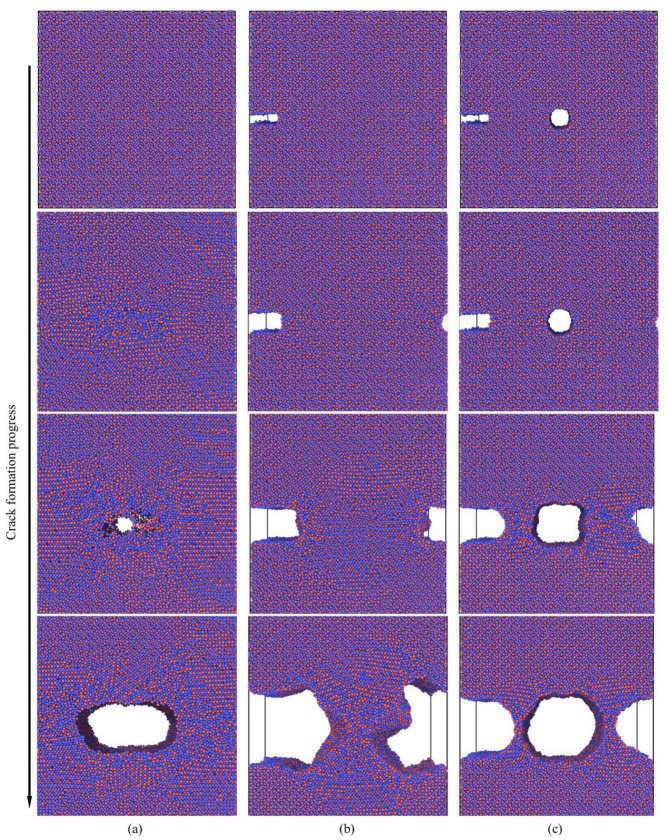
Presents the molecular dynamic simulation results for the Al_4_Cu_9_ model under different defect conditions: (**a**) non-defect; (**b**) blunt crack; (**c**) blunt crack with central void. Red represents Al, and blue represents Cu.

To analyze the impact of various defect conditions on crack propagation, we conducted molecular dynamic (MD) simulations on three models at a temperature of 300 K, as illustrated in Figure 10. The simulations reveal the initial and crack propagation stages following the increase in tensile stress. Figure 10a illustrates the tensile fracture behavior of a model without pre-existing defects, where dislocations initially occur at the central region during the early damage stage. As the tensile stress increases, these dislocations accumulate, forming voids that gradually evolve into microcracks. Dislocations accumulate significantly on both sides of the microcrack, leading to blunting at the crack tips and initiating lateral expansion. In contrast, Figure 10b,c depict scenarios where initial cracks appear near defects. As the blunt crack begins to propagate, dislocations accumulate at the right boundary of the blunt crack. The increasing dislocation density results in significant stress concentration, eventually activating nearby dislocation sources and causing the blunt crack to extend across the model. Concurrently, the left side of the blunt crack begins to expand, forming a regular path. When defects include both a blunt crack and a central void, we observe that the void enlarges into a microcrack, while the emitted dislocations slow down the interface expansion of the blunt crack. This simulation demonstrates the substantial influence of defects on dislocation movement.

**Figure 11:**
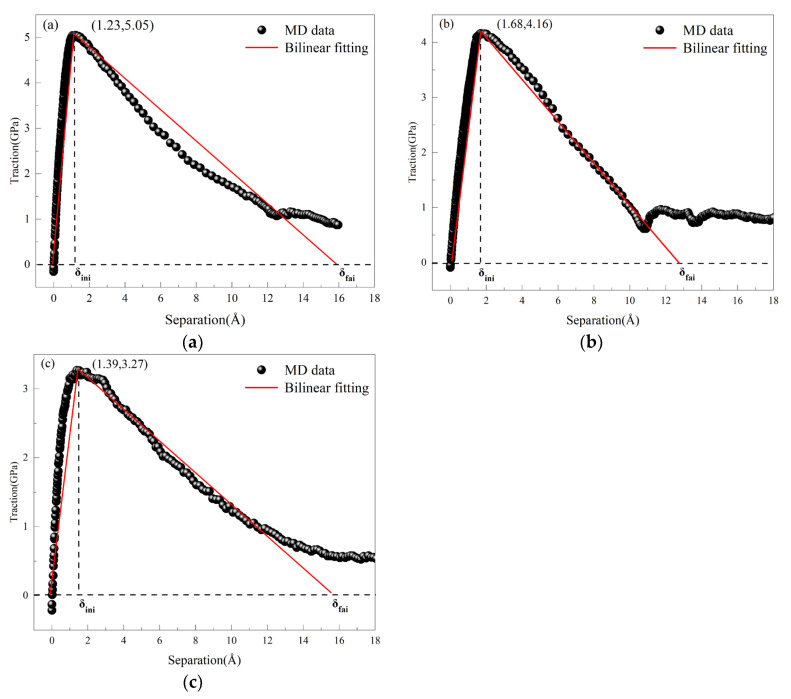
Shows the bilinear fitting T-S curves under different defect conditions: (**a**) non-defect; (**b**) blunt crack; (**c**) blunt crack with central void.

In the vicinity of defects, a traction-separation (T-S) region is established, where the average stress serves as the traction force and the average displacement as the separation displacement. This data is converted into scatter plots and fitted into bilinear fits [44,45,46], as shown in Figure 11, which displays the bilinear fitting results of the T-S curves, identifying key parameters such as maximum traction, maximum separation displacement, and stiffness coefficient. The cohesive element parameters obtained from the MD simulations are presented in Table 3. The fracture energy under different fracture conditions of the three models can be calculated using specific formulas. The table indicates that in the absence of pre-existing defects, the maximum damage stress is 5.05 GPa. This suggests that the absence of defects increases the interfacial fracture energy, requiring more energy to drive crack propagation. Conversely, when defects are present at the interface, the energy required for crack propagation decreases, making cracks more prone to expansion. Data obtained from the three different simulation methods indicate that dislocation movement is a critical factor influencing the strength of metallic materials. When dislocations encounter fewer obstacles, the material’s strength is higher. As the initial defects at the Al_4_Cu_9_ alloy interface increase, both the maximum traction and damage displacement decrease, reflecting a reduction in the material’s interfacial tensile strength.

#### 3.1.2. Macroscopic Fracture Simulation Under Different Defect Conditions

**Figure 12:**
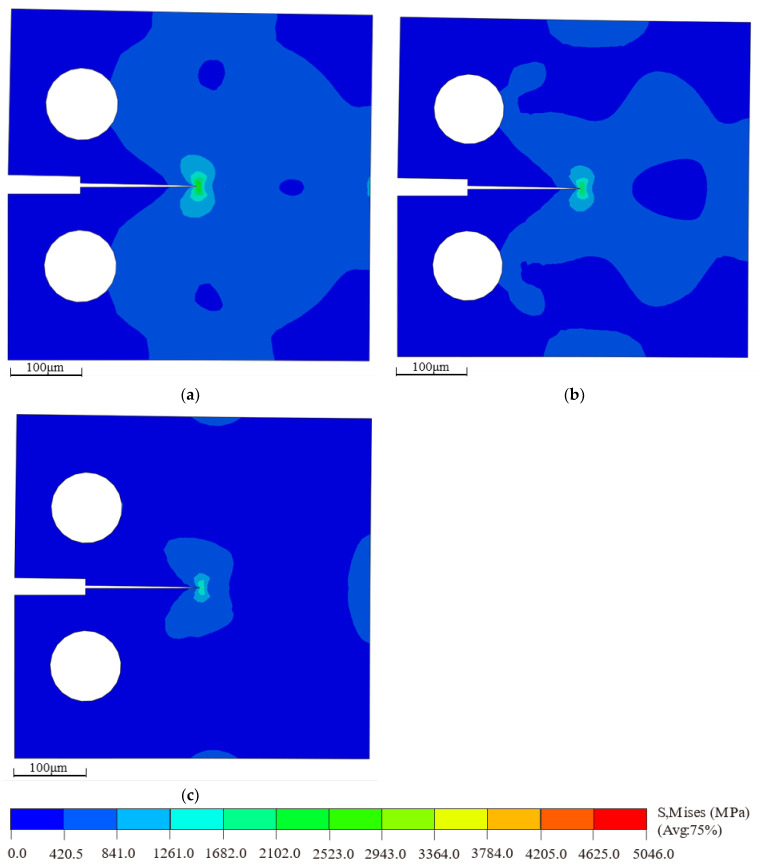
Critical stress contour plots of macroscopic compact tension specimens under different defect conditions: (**a**) non-defect; (**b**) blunt crack; (**c**) blunt crack with central void.

The bilinear cohesive force parameters under various defect conditions were input into ABAQUS to simulate the multiscale behavior of the cohesive model. The resulting critical stress contour plots are shown in Figure 12. By applying displacement loading at RP-1, the crack propagation along the predefined cohesive zone can be observed. As the displacement load reaches a critical point, the crack path begins to fully extend, and the stress redistributes around the crack tip. It is evident that the maximum Mises stress in the defect-free model, shown in Figure 12a, is higher than that in the models with defects, as shown in Figure 12b,c.

**Figure 13:**
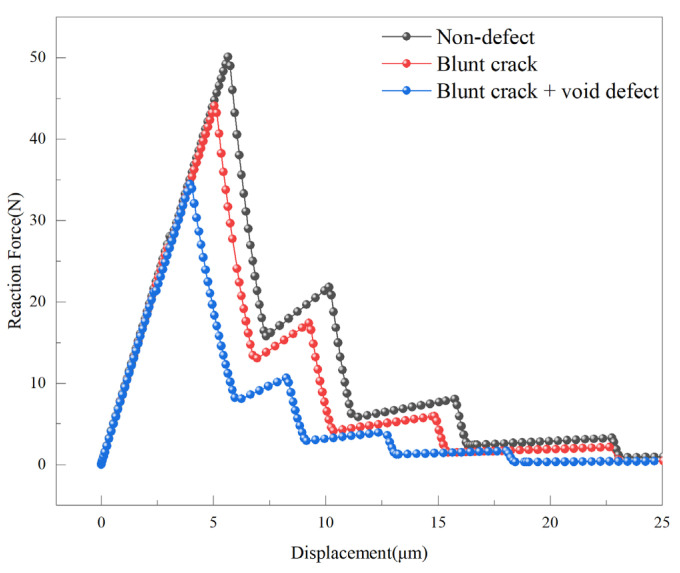
Tensile displacement-reaction force curves for compact tension specimens under different defect conditions.

The variation in the reaction force with tensile displacement for CT specimens under different defect conditions is illustrated in Figure 13. During the early elastic phase of the model, the presence of a prefabricated crack results in similar mechanical behavior across all compact tension specimens. As the displacement load increases, the crack propagates from the prefabricated crack zone into the cohesive zone. When the reaction force reaches its maximum, cohesive elements begin to degrade, initiating crack propagation.

According to ASTM E399 [40], which outlines the standard for linear elastic plane strain fracture toughness of metallic materials, the stress intensity factor at the crack tip for compact tension specimens can be expressed as:

(6) $$K_{1}=\frac{F}{B\sqrt{W}}f\left(\frac{a}{W}\right)$$

where F is the maximum reaction force applied in the Y-direction at the reference point (RP-1), W is the distance from the centerline of the loading hole to the right side of the specimen, and a is the prefabricated crack size.

Here, f(a/W) is a geometry correction function accounting for the finite specimen size effect, expressed as:

(7) $$f\left(\frac{a}{W}\right)=\left(\frac{2+\sqrt{\frac{a}{W}}}{{\left(1-\frac{a}{W}\right)}^{3/2}}\right)\cdot \left[0.886+4.64\left(\frac{a}{W}\right)-13.32{\left(\frac{a}{W}\right)}^{2}+14.72{\left(\frac{a}{W}\right)}^{3}-5.60{\left(\frac{a}{W}\right)}^{4}\right]$$

Considering that thickness effects can be neglected under 2D plane strain conditions, the specimen thickness B is chosen to be 1 micron. A similar treatment has been adopted in previous studies [47], indicating that this thickness choice has negligible effect on the results, and a sensitivity analysis is not required.

The displacement-reaction force relationship obtained from finite element simulations is used to calculate the stress intensity factor, which depends on the critical stress at the onset of material failure. The critical stresses for the defect-free model, blunt crack model, and blunt crack with void model are 50.11 N, 44.10 N, and 34.43 N, respectively. The maximum stress intensity factors are 24.21 MPa∙m^1/2^, 21.30 MPa∙m^1/2^, and 16.63 MPa∙m^1/2^, respectively. The stress intensity factors have an uncertainty of 1–2%.

These results indicate that defect type significantly influences the stress intensity factor: the blunt crack with void defect reduces the stress intensity factor by 31.31%, compared to a reduction of 12.02% for the single blunt crack. This reduction arises from the damage competition mechanism, which refers to the interaction between the crack tip and the local stress fields induced by voids and second-phase particles. The existence of voids introduces local free surfaces and causes stress concentration along the void boundary, thereby facilitating earlier crack propagation and reducing the overall tensile strength. Similar mechanisms were observed in molecular dynamic simulations of Al_2_Cu, where increasing void size and density significantly decreased the alloy’s tensile strength and ductility due to intensified stress localization near the void edges [48]. This stress redistribution and interaction between micro-defects and the crack front jointly govern the observed decrease in the effective stress intensity factor.

## 4. Discussion

### 4.1. Analysis of Crack Propagation Behavior Under Different Temperature Conditions

#### 4.1.1. Microscopic Fracture Simulation Under Different Temperature Conditions

**Figure 14:**
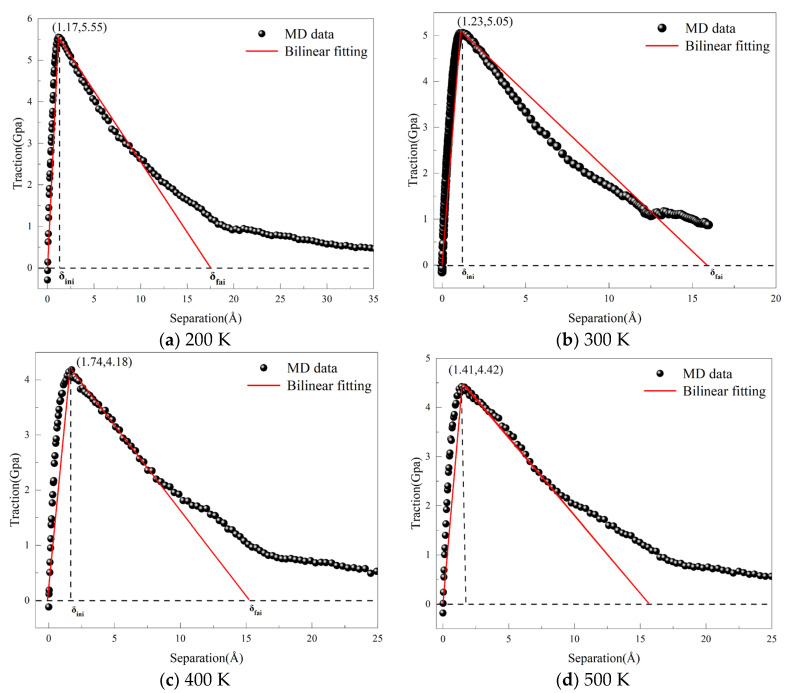
Bilinear Fitting T-S Curves of the Al_4_Cu_9_ Model at Different Temperatures.

According to the European Aerospace Standard EN 4681 [49] and the Chinese GB/T 42043-2022 standard [50], which outline the general technical specifications for copper-clad aluminum wire cables, the rated operating temperature for this type of cable is −65 °C to 180 °C. To investigate the impact of temperature on the tensile deformation mechanical properties of the Al_4_Cu_9_ alloy, simulations were conducted on a defect-free model at four temperatures: 200 K, 300 K, 400 K, and 500 K. Other simulation parameters remained constant. The simulation employed a microcanonical ensemble (NVE) and ran for 50,000 steps, outputting coordinate information, temperature, stress, and displacement every 300 steps. The bilinear fitting T-S curves corresponding to different temperatures are shown in Figure 14.

Figure 14 further elucidates the regulatory effect of temperature on the damage evolution process of Al_4_Cu_9_. The bilinear cohesive force parameters under different temperature conditions are presented in Table 4. As the temperature increases from 200 K to 500 K, the fracture energy decreases from 4.80 J/m^2^ to 3.72 J/m^2^, and the strength decreases from 4.74 × 10^7^ μN/μm^3^ to 2.40 × 10^7^ μN/μm^3^. The simulations reveal that increasing temperature markedly alters the dislocation activity at the crack tip. At 200 K, insufficient thermal activation limits dislocation nucleation, and the crack propagates rapidly along crystallographic planes with lower atomic packing density, exhibiting typical brittle fracture characteristics. At 500 K, thermal activation promotes continuous dislocation nucleation and glide near the crack tip, with some dislocations interacting and entangling at the interface, producing local stress-shielding effects that lead to crack deflection or blunting. Moreover, the mismatch in thermal expansion coefficients between the Al_4_Cu_9_ phase and the matrix at elevated temperatures induces a redistribution of interfacial stresses, and enhanced atomic diffusion increases the likelihood of crack propagation along defect-rich or dislocation-dense regions. These dislocation and microstructural evolution mechanisms account for the observed temperature dependence of fracture energy and crack path selection.

#### 4.1.2. Macroscopic Fracture Simulation Under Different Temperature Conditions

**Figure 15:**
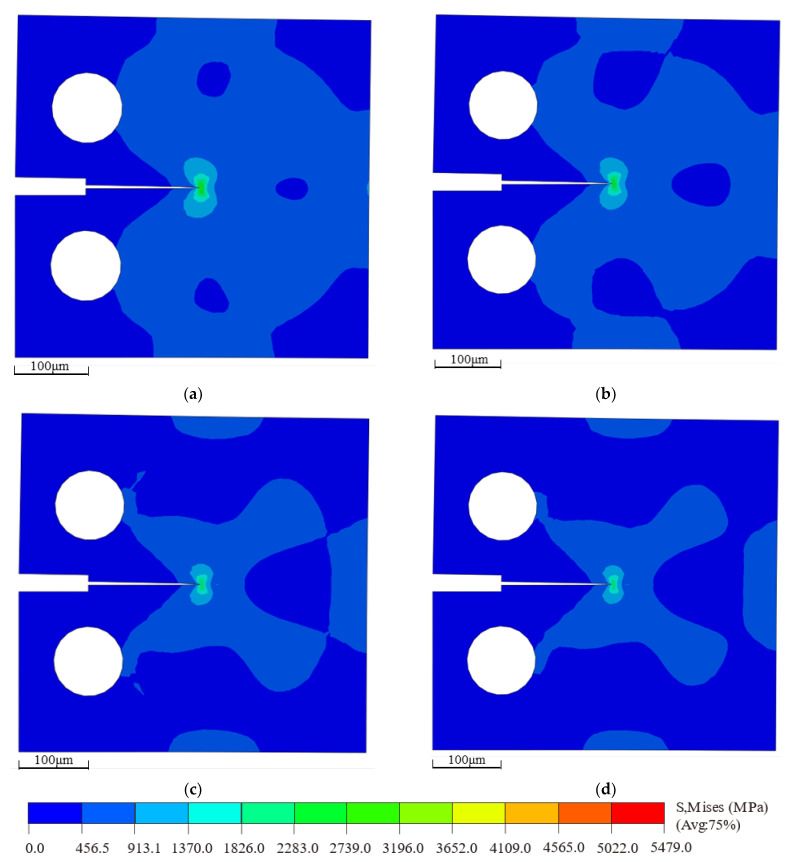
Critical Stress Contour Plots of Macroscopic Compact Tension Specimens at Different Temperatures: (**a**) 200 K; (**b**) 300 K; (**c**) 400 K; (**d**) 500 K.

The bilinear cohesive zone parameters under different temperature conditions were input into ABAQUS to simulate the multiscale behavior of the cohesive zone model. The resulting critical stress contour plot is shown in Figure 15. By applying displacement loading at RP-1, the crack propagation along the designated cohesive region can be observed. When the displacement load reaches the critical point, the crack path exhibits a stable expansion mode, and the stress field diffuses outward. It is evident that the stress field at the crack tip diminishes with increasing temperature, indicating that within the 200 K–500 K range, the maximum Mises equivalent stress at the critical point decreases with rising temperature.

**Figure 16:**
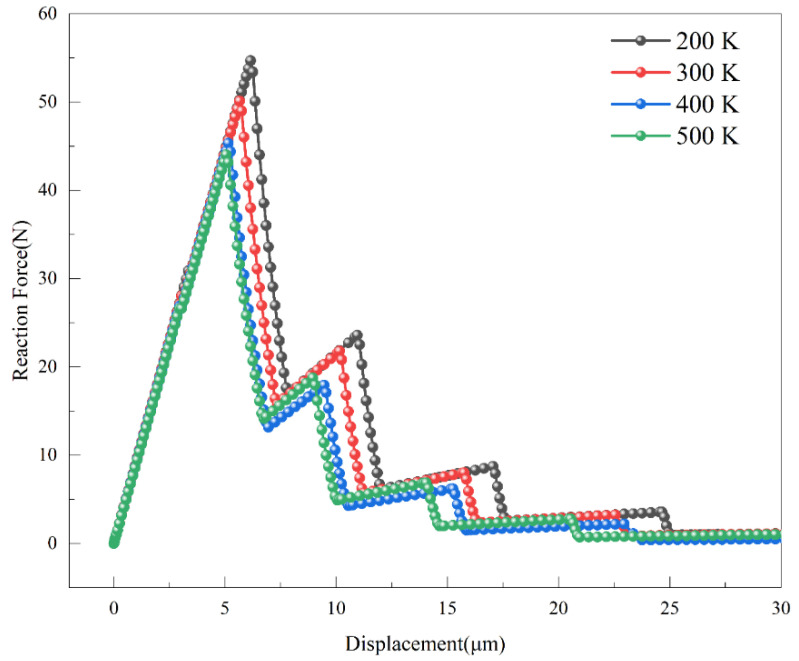
Tensile Displacement-Support Reaction Force Curves of Compact Tension Specimens Under Different Temperature Conditions.

The variation in the support reaction force with time for crack propagation in CT specimens under different temperature conditions is depicted in Figure 16. The critical stresses at 200–500 K are 54.70 N, 50.11 N, 45.34 N, and 44.06 N, respectively. Within the 200–500 K range, the maximum support reaction force of the entire model decreases with increasing temperature, while the tensile displacement shows a slight increase. The maximum stress intensity factors for cracks at 200–500 K, calculated using Equation (6), are 26.42 MPa∙m^1/2^, 24.21 MPa∙m^1/2^, 21.90 MPa∙m^1/2^, and 21.28 MPa∙m^1/2^, respectively. The uncertainty of the calculated stress intensity factors, estimated based on mesh sensitivity and repeated simulation runs, is within 1–2%.

### 4.2. Limitations and Future Work

#### 4.2.1. Macroscopic Fracture Simulation: Limitations and Future Work

The models primarily employed uniaxial tensile loading and considered only a representative interface orientation, which restricts the generality of the extracted cohesive parameters. Future work should incorporate more diverse interface orientations and loading modes to establish a more comprehensive parameter database.

#### 4.2.2. Material Anisotropy

Although Al_4_Cu_9_ crystallizes in a cubic system, its fracture toughness and dislocation mobility may still exhibit orientation dependence. The present work did not systematically address this anisotropy, and the potential influence of orientation-dependent toughness on the results warrants further investigation.

#### 4.2.3. Future Research

At the meso- and macro-scales, the simulations focused mainly on opening-mode cracks, while shear-mode fracture was not explicitly considered. Future studies should integrate microscale shear cohesive parameters into multiscale simulations to achieve a more comprehensive description of intergranular fracture in polycrystalline materials.

## 5. Conclusions

1. A multiscale MD–CZM–FE framework was established, quantitatively linking atomistic dislocation evolution with macroscopic fracture toughness through traction–separation parameters.
2. Defect type exerts a dominant effect: a composite defect (blunt crack + void) reduces fracture energy by 39.95% and SIF by 31.31%, compared with 31.27% and 12.02% for a single blunt crack.
3. The temperature rise from 200 K to 500 K decreases fracture energy (−22.5%) and SIF (−19.5%), due to enhanced dislocation activity, stress redistribution, and diffusion-induced crack deflection.
4. Cross-scale simulations show that defect effects (18.4% variation) outweigh thermal effects (10.0%). Experimental crack initiation sites agree with predictions, validating the framework’s reliability.
5. Beyond academic significance, the proposed framework provides a potential tool for reliability assessment of Cu/Al conductors in aerospace electrical systems, offering guidance for structural design and service-life prediction.

## Figures and Tables

**Table 1:** Comparison of calculated and reported mechanical parameters of Al_4_Cu_9_.

Parameter	Elastic Constants			Lattice Constant (Å)	Cohesive Energy (eV)	Bulk Modulus (GPa)
	C_11_ (GPa)	C_12_ (GPa)	C_44_ (GPa)			
Experimental [35]	223.4	122	85	8.706	−4.0747	155.7
This work (calculated)	208	131	53	8.816	−3.633	153.1

**Table 2:** Mapping of MD Parameters to ABAQUS (mm unit system).

MD Parameter	MD Unit	ABAQUS Parameter	ABAQUS Unit (mm Unit System)	Conversion
$\mathrm{Damage}\;\mathrm{Stress}\;(N_{\max}$)	GPa	$\mathrm{Nominal}\;\mathrm{stress}\;(\sigma_{nn}$)	MPa (N/mm^2^)	$\sigma_{nn}\;=N_{\max}\times 10^{3}$
Strength (K)	μN/μm^3^	$\mathrm{Elastic}\;(E_{nn}\;$)	N/mm^3^ (MPa/mm)	$E_{nn}\;=K\times 10^{3}$
$\mathrm{Fracture}\;\mathrm{Energy}\;(G_{c}$)	J/m^2^	$\mathrm{Fracture}\;\mathrm{energy}\;(G_{c}^{FE}$)	N/mm	$G_{c}^{FE}=G_{c}\times 10^{-3}$

**Table 3:** Bilinear cohesive force parameters under different defect.

Type	Damage Stress (GPa)	Maximum Separation (Å)	Strength (10^7^ μN/μm^3^)	Fracture Energy (J/m^2^)
Non-defect	5.05	15.98	4.11	4.03
Blunt crack	4.16	13.25	2.47	2.77
Blunt + void defect	3.27	14.83	2.35	2.42

**Table 4:** Bilinear cohesive force parameters under different temperature conditions.

Temperature	Damage Stress (GPa)	Maximum Separation (Å)	Strength (10^7^ μN/μm^3^)	Fracture Energy (J/m^2^)
200 K	5.55	17.31	4.74	4.80
300 K	5.05	15.98	4.11	4.03
400 K	4.42	17.61	3.13	3.89
500 K	4.18	17.80	2.40	3.72

## Data Availability

The raw data supporting the conclusions of this article will be made available by the authors on request.

## Abbreviations

The following abbreviations are used in this manuscript:

MD	Molecular Dynamics	$N_{\max}$	Maximum Interface Traction
FE	Finite Element	$\delta_{\mathrm{ini}}$	Initial Displacement of Interface
CZM	Cohesive Zone Model	$\delta_{fai}$	Failure Displacement of Interface
T-S	Traction–Separation	K	Initial Stiffness
EAM	Embedded Atom Method	$K_{n}$	Normal Stiffness
CT	Compact Tension	$K_{s}$	Shear Stiffness
NPT	Isothermal–Isobaric Ensemble	$K_{t}$	Tangential Stiffness
NVE	Microcanonical Ensemble	W	Width of CT Specimen
PBCs	Periodic Boundary Conditions	a	Initial Crack Length
OFHC	Oxygen-Free High-Conductivity copper	$a_{0}$	Lattice Constant
OM	Optical Microscopy	F	Maximum Reaction Force
SEM	Scanning Electron Microscopy	B	Thickness of CT Specimen
ASTM	American Society for Testing and Materials	$K_{\mathrm{IC}}$	Fracture Toughness/Stress Intensity Factor
E	Cohesive energy/Potential energy per atom	$V_{0}$	Equilibrium unit cell volume
$B_{0}$	Bulk modulus at equilibrium	$G_{c}$	Fracture energy (in MD)
M	Number of atoms in the unit cell	$G_{c}^{FE}$	Fracture energy (in ABAQUS)
$C_{11}$	Longitudinal elastic constant	$\sigma_{nn}$	Damage Initiation (in ABAQUS)
$C_{12}$	Coupling elastic constant	$E_{nn}\;$	Elastic–Traction (in ABAQUS)
$C_{44}$	Shear elastic constant		
